# Supplementary figures and images for: Association of Tandem Repeat Number Variabilities in Subunit S of the Type I Restriction-Modification System with Macrolide Resistance in Mycoplasma pneumoniae
Source: J Clin Med. 2022 Jan 28;11(3):715. doi: 10.3390/jcm11030715 (PMC8836594; doi:10.3390/jcm11030715)

## Slide 1
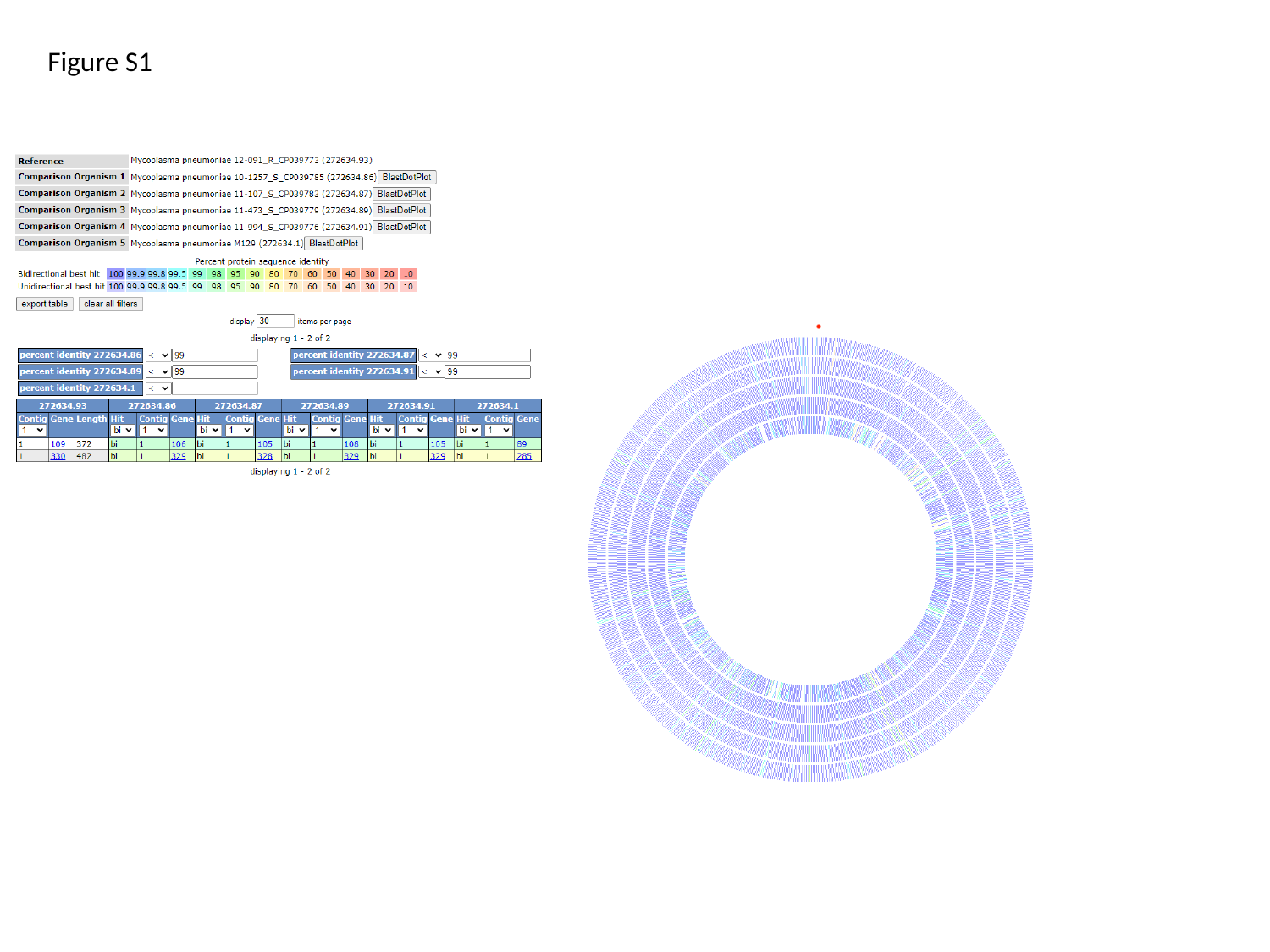

Figure S1

## Slide 2
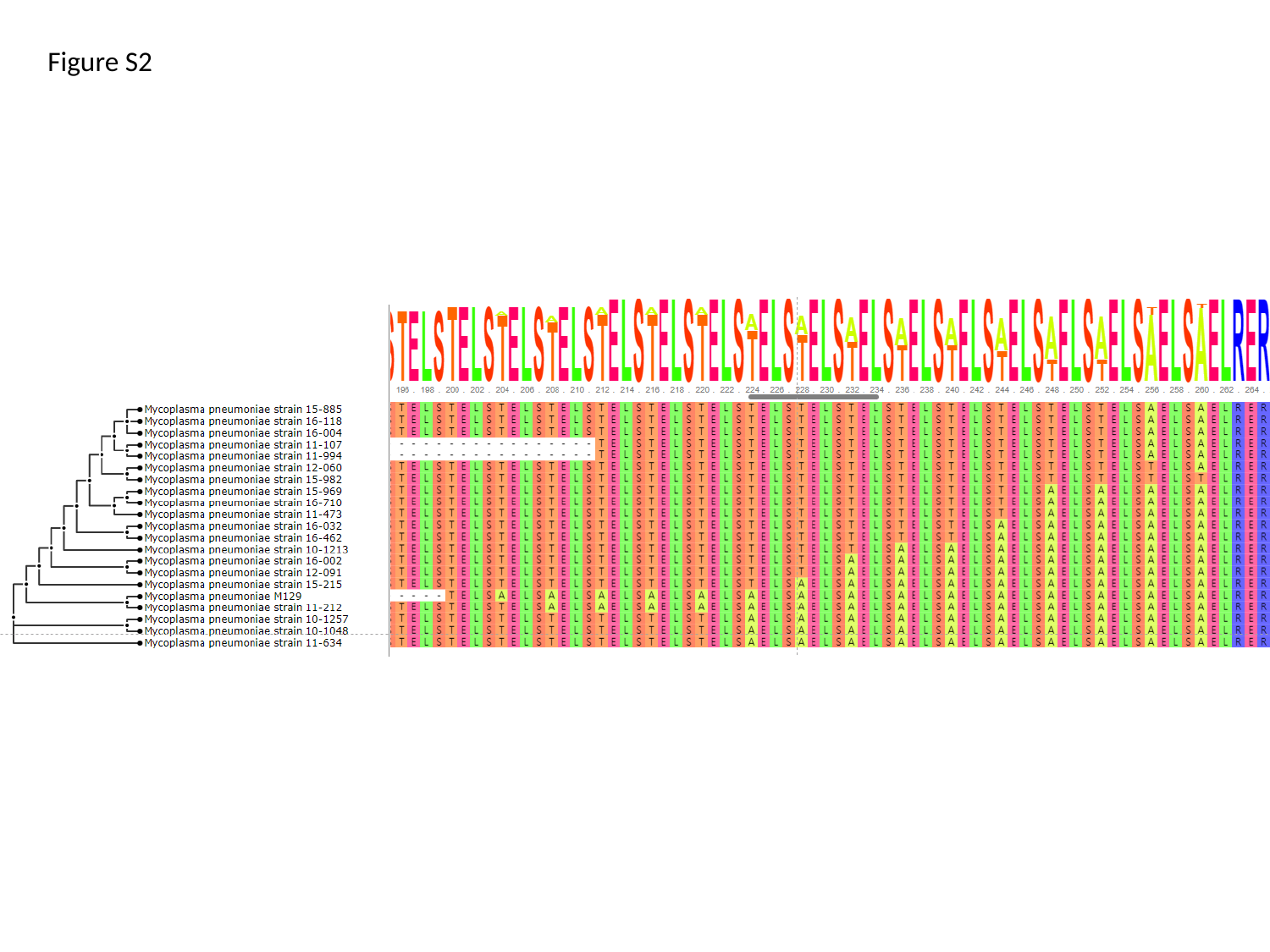

Figure S2

## Slide 3
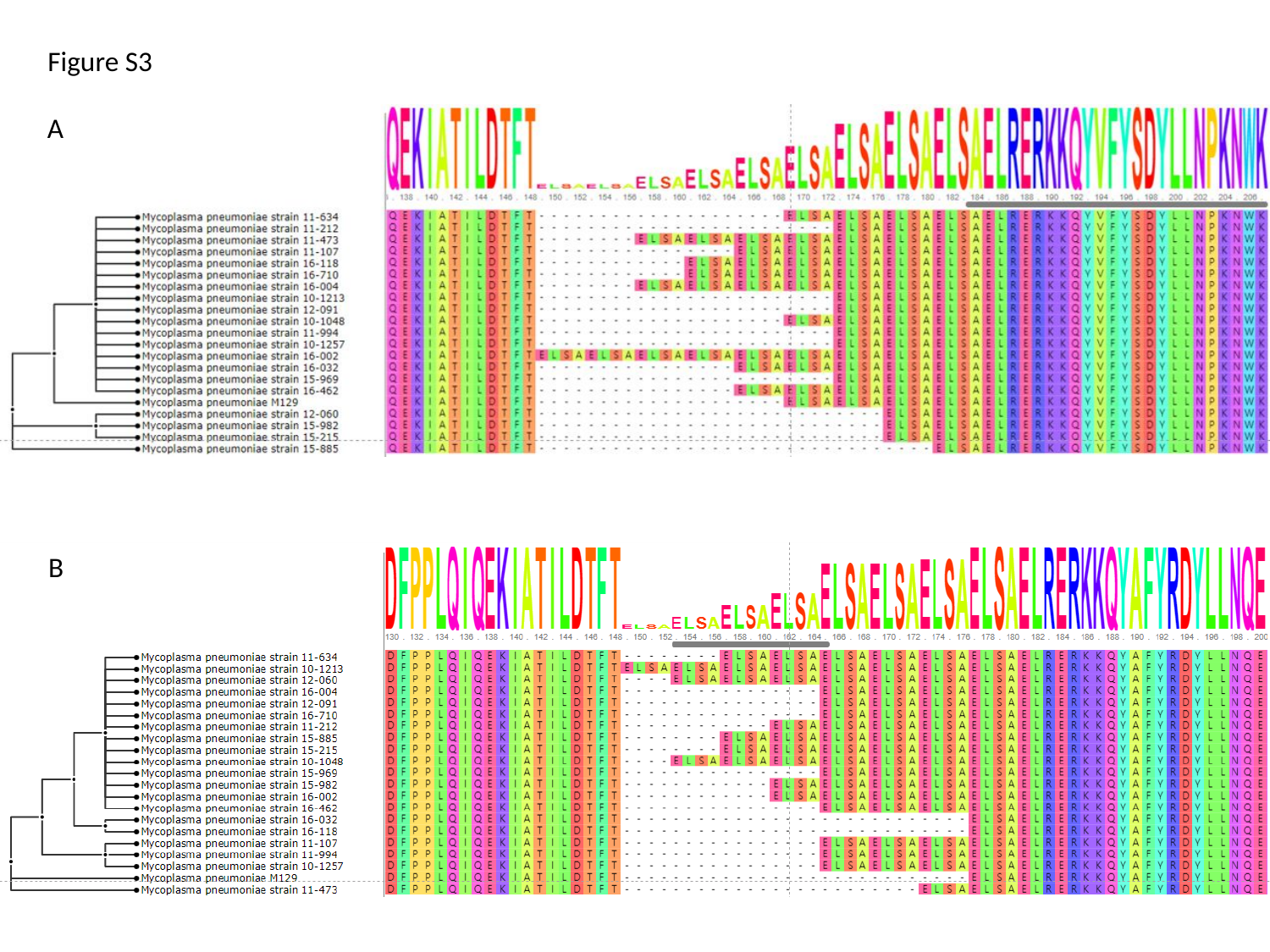

Figure S3
A
B

Supplement: Supplementary file 1 [file jcm-11-00715-s001.zip › Tandem repeat number variabilities-supplementary_figure.pptx]
